# From Shorter to Longer Timescales: Converging Integrated Information Theory (IIT) with the Temporo-Spatial Theory of Consciousness (TTC)

**DOI:** 10.3390/e24020270

**Published:** 2022-02-13

**Authors:** Georg Northoff, Federico Zilio

**Affiliations:** 1 Royal Ottawa Mental Health Centre, Mind, Brain Imaging and Neuroethics Research Unit, Institute of Mental Health, University of Ottawa, Ottawa, ON K1N 6N5, Canada; 2 Department of Philosophy, Sociology, Education and Applied Psychology, University of Padua, 35139 Padua, Italy; federico.zilio@unipd.it

**Keywords:** integrated information theory, temporo-spatial theory of consciousness, temporal integration, pre-stimulus activity, resting state, task-related activity, phenomenal content, contents of consciousness, stream of consciousness

## Abstract

Time is a key element of consciousness as it includes multiple timescales from shorter to longer ones. This is reflected in our experience of various short-term phenomenal contents at discrete points in time as part of an ongoing, more continuous, and long-term ‘stream of consciousness’. Can Integrated Information Theory (IIT) account for this multitude of timescales of consciousness? According to the theory, the relevant spatiotemporal scale for consciousness is the one in which the system reaches the maximum cause-effect power; IIT currently predicts that experience occurs on the order of short timescales, namely, between 100 and 300 ms (theta and alpha frequency range). This can well account for the integration of single inputs into a particular phenomenal content. However, such short timescales leave open the temporal relation of specific phenomenal contents to others during the course of the ongoing time, that is, the stream of consciousness. For that purpose, we converge the IIT with the Temporo-spatial Theory of Consciousness (TTC), which, assuming a multitude of different timescales, can take into view the temporal integration of specific phenomenal contents with other phenomenal contents over time. On the neuronal side, this is detailed by considering those neuronal mechanisms driving the non-additive interaction of pre-stimulus activity with the input resulting in stimulus-related activity. Due to their non-additive interaction, the single input is not only integrated with others in the short-term timescales of 100–300 ms (alpha and theta frequencies) (as predicted by IIT) but, at the same time, also virtually expanded in its temporal (and spatial) features; this is related to the longer timescales (delta and slower frequencies) that are carried over from pre-stimulus to stimulus-related activity. Such a non-additive pre-stimulus-input interaction amounts to temporo-spatial expansion as a key mechanism of TTC for the constitution of phenomenal contents including their embedding or nesting within the ongoing temporal dynamic, i.e., the stream of consciousness. In conclusion, we propose converging the short-term integration of inputs postulated in IIT (100–300 ms as in the alpha and theta frequency range) with the longer timescales (in delta and slower frequencies) of temporo-spatial expansion in TTC.

## 1. Introduction

### 1.1. IIT and Its Axioms—Phenomenal Content and Its Temporal Characterization

How does the inner activity of a system, like the neuronal activity of the brain, transform into the experience of phenomenal contents in our ongoing consciousness? One of the most relevant neuroscientific theories of consciousness, Integrated Information Theory (IIT), claims that if a system has the intrinsic potential to integrate information, then we can assume the presence of consciousness [1,2,3,4,5]. This system can be the brain but also other physical substrates that present the same kind of causal structure. IIT promises great explanatory, predictive, and inferential power to determine whether, how much, and in which way a system is conscious [3]. To link information integration to the phenomenal realm of experience, Tononi and colleagues define five axioms about experience that should be self-evident, essential, complete, consistent, and independent [1,2,5]:

- Intrinsic existence: any experience is real and exists intrinsically, and I am immediately sure of it.
- Composition: any experience is structured into various parts, qualities, and objects.
- Information: any experience is composed of a specific set of information that differs from other possible sets.
- Integration: any experience is a unitary whole that is irreducible to its independent components.
- Exclusion: any experience is specified on a single set of elements and is definite in content and spatio-temporal grain, thus it excludes other possible experiences at the same time.

These phenomenological axioms are translated into postulates of the physical substrate of consciousness. It is important to analyze the identity between integrated information and experience: if having an experience means being conscious, and if integrated information is a kind of causal property within a physical system, it is possible to translate the axioms of experience into postulates of the physical substrate of consciousness (PSC). Experience is the capacity of a system to integrate information, that is, to discriminate among a set of possible states. The object of analysis, description, and prediction of IIT is essentially the phenomenal content at a particular discrete point in time that holds over a short timescale, namely 100 to 300 ms as predicted by IIT. Indeed, IIT currently predicts that the maximum intrinsic cause-effect power of the PSC must be reached at that timescale rather than on the order of microseconds or multiple seconds [1,6,7]. However, beyond that rather discrete timescale of 100–300 ms (theta and alpha frequency range), our experience, in addition to including discrete elements [8,9,10], also appears to be composed of longer-term features that work more continuously [11,12,13,14,15,16,17]: this is, for instance, reflected in the embedding or nesting of short-term phenomenal contents within longer timescales (with delta and slower frequencies) of the ongoing stream of consciousness [13,15,18,19,20].

What provides the more continuous long-term temporal link between the single more discrete short-term phenomenal contents resulting in the experience of a stream of consciousness? Addressing this question, we propose converging the IIT with its focus on short-term integration over 100–300 ms with the Temporo-spatial Theory of Consciousness (TTC) [21,22,23] as that includes multiple timescales both short- and long-term (i.e., fast and slow frequencies).

### 1.2. IIT—Short Timescales (100–300 ms) vs. Longer Timescales in Consciousness

Tononi et al. (2016) argue that the instant of experience shows a defined duration, especially since the PSC is defined by the biological conditions of the brain [1]. This ‘defined’ duration is actually variable and ranges from about ten milliseconds to a few hundred milliseconds; it falls within a specific time scale, excluding timescales that are too small (microseconds) and too long (seconds and minutes) [1,6,7]. Although, in principle, all timescales are considered by IIT (particularly in early versions of IIT [12,24]), the variability in milliseconds (100–300 ms) of experience depends on which of these timescales the system reaches the maximum cause-effect power. Tononi et al. draw this conclusion from the duration of experience from a first-person perspective, as it does not seem possible to have a content of microseconds or many seconds or even minutes [1,25]. However, there is no consensus on the duration of a single phenomenal content, rather several recent studies argue for a variety of different durations in our experience of phenomenal content, including also longer-term durations such as three seconds and more [12,17,26,27,28,29,30].

Yet another point is the integration between different contents. Miyahara and Witkowski [31] point out that IIT is exclusively concerned with the phenomenal content of consciousness, so integration occurs at the level of the content components of the cause-effect structure but not among the sequence of discrete experiences themselves. In other words, integration in IIT happens within one momentary experience and not across experiences. In fact, the theory seems to assert that phenomenal contents follow one after the other in a more or less juxtaposed way, depending on which complex beats the others by generating a local maximum of integrated information within the updated neural substrate: As the substrate changes (more or less gradually), the phenomenal contents will change accordingly, providing some degree of continuity of experience. On the contrary, the possibility of overlap or embedding of different phenomenal contents—and relative cause-effect structures—appears to be absent on longer timescales beyond the short-term ones (100–300 ms) predicted by IIT [1,5,7,32]. It is in fact more plausible to represent the experience according to IIT as a movie-like sequence of independent frames (i.e., every single cause-effect structure) juxtaposed one after the other.

The IIT itself provides an indirect hint about the relation between different phenomenal contents. In the axioms it is stated that the components of the phenomenal contents have their own identity from their interconnections within the integrated whole by the integration axiom [31]: it is not possible to reduce the perception of a blue book by separating the components into ‘book without the color blue’ + ‘color blue without the book’ [5]. However, in the same way, one cannot separate the phenomenal content from the other contents and, more generally, from the stream of consciousness [33]. Briefly, the contents of consciousness cannot be separated from the other phenomenal contents of the ongoing stream of consciousness.

Taken together, there may be a temporal gap between the short-term timescales (100–300 ms) predicted by IIT for the constitution of phenomenal contents and our experience of temporal relation among different phenomenal contents over longer-term timescales, i.e., stream of consciousness. Addressing this temporal gap is the goal of our paper.

What kind of timescales are required to close this temporal gap? For example, the contents of consciousness with defined duration can derive their meaning from their mutual integration within longer acts of consciousness and at different timescales of the stream. In other words, the apprehension of a sentence may require not only a few milliseconds of awareness of individual words but also several seconds until the last word is processed [26,34]. As argued by Herzog et al. [9], the specific meaning of sentences such as ‘The mouse in my office is broken’ and ‘The mouse in my office is dead’ is determined by the last word. Importantly, this is not just a matter of semantic memory, since the phenomenal content of ‘the mouse’ is also determined by the last words: in the first case, I mentally visualize the computer mouse, in the second the animal—this makes it clear that the context in which the phrase is heard shapes its meaning and the way in which I experience the words.

Accordingly, it seems that IIT does not consider longer-term timescales that make possible the temporal integration of phenomenal contents in a diachronic sense. As formalized in Φ, integration just refers to the formal integration of information within a system at a specific time point. This involves short-term synchronicity and discreteness, while not allowing long-term diachronicity and continuity of our consciousness [11,12]. Complementing the focus on short-term timescales in IIT by longer-term timescales as a key element of the diachronic and continuous nature of the stream of consciousness is the main objective of our paper.

### 1.3. From IIT to TTC—Expanding Timescales Allow for Three Different Forms of Temporal Integration

**Figure 1:**
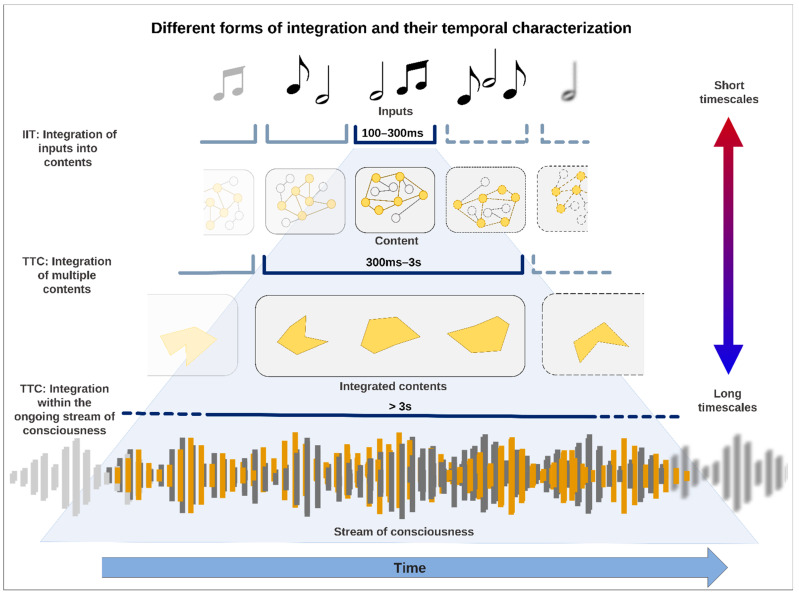
Three different forms of integration on different time scales. The shortest temporal range (≈100–300 ms) corresponds the integration of inputs (e.g., sound notes) into phenomenal contents according to IIT. The medium temporal range (≈300 ms–1 s) corresponds the phenomenal and semantic integration of different contents of consciousness (‘The mouse in my office is broken/dead’) depending on the context and background of the intrinsic activity of the brain. The longest temporal range (≈<3 s) corresponds to the integration of macro contents within the ongoing stream of consciousness that provides phenomenal duration, continuity, coherence, and unity.

We are confronted with three different forms of integration (see Figure 1). First, there is the integration of the components that characterize a phenomenal content of consciousness (100–300 ms)—IIT accounts for that by information integration together with its five axioms. Secondly, there is the integration of the phenomenal content of consciousness with other contents of consciousness as the single content is dependent upon its relation to other contents and the temporo-spatial context and background of the intrinsic activity of the brain (see below) (300 ms–3 s). This level of integration is compatible with several studies on the extent of the ‘experienced moment’ [12,17,26,27,28,30,34,35]. Finally, and third, there is the integration of phenomenal contents with the ongoing consciousness itself in a time range greater than 3 s, i.e., the stream of consciousness. Following James’ account of consciousness, who distinguishes the very contents of the experience (the ‘substantive parts’) from the transitional periods that provide the temporal link between the contents (the ‘transitive parts’) [15,33,35], the stream of consciousness is conceived here neither as additional content of consciousness nor simply as the frame-by-frame sequence of short-duration contents [26,27,36] but rather as activity that gives duration, continuity, coherence, and unity of the experience [13,14,15,37,38,39,40,41]. In this regard, it can be assumed that various timescales of integration in turn integrate and select extended chunks of experience into even longer units of experiences, in which functions such as working memory, attention, and autobiographical self support a balance between continuity of experience and change. Montemayor and Wittmann (2014) argue that mental presence can extend up to 30 s [27], while Kent (2019) and Wackermann (2007) argued that it can extend up to 100 s [30,37]. At the same time, shorter timescales (<100 ms) related to the psychological functions of temporal resolution, detection, order, synchronization, etc. could also be considered but referring to the functional moment [17,27,30]. However, for the purpose of this paper, we will focus on these levels of integration specifically related to the moments experienced and their relationship with the stream of consciousness.

The entire temporal range from a few milliseconds to 100 s is methodologically explorable by estimating the duration and characteristics of the intrinsic neural timescales (with fMRI, e.g., 0.01–0.1 Hz and EEG, e.g., 0.5–100 Hz). Recently, dynamical measurements such as the Power-law Exponent and Autocorrelation Window, which can span shorter and longer timescale intervals (even up to 100 s), have shown a repertoire of scale-free dynamics associated with various features of consciousness, e.g., wakefulness, self-consciousness, perception, input integration, and segregation [38,39,40,41]. The stream of consciousness could therefore not be linked to a specific duration but could extend over different and overlapping timescales, depending on the experiential context and the different features of consciousness involved, rather than bound only to the onset of a paradigmatic stimulus or content of consciousness. It remains to be explicitly tested which specific group of timescales is associated with the stream of consciousness.

IIT only accounts for the first type of discrete integration (i.e., the phenomenal contents follow one after the other depending on which complex beats the others by generating a local maximum of integrated information within the updated neural substrate [1,5,6,7]). Instead, the continuity of experience and the temporal extension, rather than being understood as intrinsic features of consciousness, are assumed to be determined by the similarity of subsequent PSCs. In contrast, it seems to leave open both the second and third types, i.e., the integration of different phenomenal contents and their integration within the stream of consciousness. IIT has a mechanistic view that focuses on neurons, their connections, and their current activity within the current experience, which is necessarily determined both from within (the cause-effect structure itself) and from the outside by the background conditions, which indirectly shape and influence the content of consciousness, e.g., sensory inputs, activity from subcortical regions, resting-state activity, neuromodulators, etc. [1,2,42]. TTC does not consider, as a starting point, neuronal activity and its relationship among different regions at a definite moment in time; rather, it stresses the relevance of the temporal dynamics of neuronal activity at different points in time as an intrinsic feature of consciousness [21,23,43,44]. This temporal dimension can be conceived as an empirical specification of the background conditions that influence the shape of current activity in the cause-effect structure of the PSC predicted by IIT [32,45].

We here assume that the three different types of integration can be featured by different timescales along the spectrum of shorter to longer ones—they are thus distinct forms of temporal integration. As said, IIT predicts that the relevant timescales for the constitution of an ‘instant’ of experience are between 100 and 300 ms [7], which may be ideal for integrating different inputs into one content, that is, phenomenal content. However, this falls short of accounting for the longer timescales that are needed for the second and third forms of integration. The second form of integration, namely the integration between different contents, may range beyond 300 ms up to one second. Although the integration of phenomenal contents within the ongoing stream of consciousness may recruit even longer timescales, the actual phenomenal content is just a brief moment within the continuously ongoing stream of consciousness that provides the phenomenal context of our experience. To account for the three types of temporal integration, IIT requires an extension of its short-term timescales (100–300 ms as reflecting theta and alpha frequency range) by longer-term timescales (>300 ms as going down to delta and slower frequencies). Such a multitude of short- and long-term timescales is one key element of the Temporo-spatial Theory of Consciousness (TTC) [21,22,23,46]. In summary, TTC assumes that the brain’s constructions of its own inner time and space involve a multitude of different short- and long-term timescales that, involving the three forms of temporal integration, are all involved in constituting different phenomenal layers or features of consciousness.

The TTC assumes three-fold temporal integration of (i) inputs into contents, (ii) contents with contents, and (iii) contents with the stream of consciousness. How does such a three-fold temporal integration occur? For that, the TTC assumes the interaction between pre-stimulus activity and input to be the key—this interaction is driven by a particular mechanism, temporo-spatial expansion, that is essentially based on integrating different temporal (and spatial) scales including shorter and longer ones. Our paper focuses on that mechanism, the temporo-spatial expansion (which is one of the four temporo-spatial mechanisms postulated in TTC). In the first part, we explain the neuronal side of this mechanism including its relation to conscious contents, while in the second part we focus on its neuro-phenomenal relevance, that is, its importance for the phenomenal features of consciousness.

Furthermore, among the three types of integration, in particular between the first (main focus of IIT) and the other two (main focus of TTC), there is not only a quantitative difference based on the temporal length of the integration processes. The temporal timescale of 100–300 ms predicted by IIT is a contingent consequence of the maximum of intrinsic cause-effect power reached; in contrast, TTC conceives of the temporal extension of the other two types of integration and the possibility of multiple overlapping timescales as a fundamental feature of the integration of various contents of consciousness. This difference can be explained mathematically through the category theory framework [43]. Considering two neuronal categories, N0 and N1 and their relationships, according to IIT, N0 is the actual state of the neural activity of a network at a given moment, and N1 is all possible states of the neural activity of all possible networks at the same moment. IIT argues that what directly determines the content-specific neural correlate of consciousness is not the activity of N0 alone but N0 in relation to all possible activities of N1 (but within N0). In other words, the conscious content is such (informative) because it specifies one of all possible experiences, in the same way in which N0 (the actual neural state) is a specification of all possible configurations expressed by N1. Instead, TTC considers N0 and N1 not as the logical difference between actual and possible states but as a difference of temporal order, that is, the dynamics of neuronal activity as it operates across different regions and points in time. In this case, N0 precedes N1 in time, that is, N0 is regarded as the pre-stimulus activity, while N1 is regarded as post-stimulus activity, where the relationship is constituted by the non-additive interaction between the two categories, which is associated with the content-specific NCC. In this sense, TTC extends the concept of experience beyond a single actual neuronal state by assuming the temporo-spatial dynamics of two distinct neuronal states necessary to underlie consciousness. Therefore, while IIT claims that consciousness comes with the phenomenal content itself, TTC claims that consciousness is the interaction between pre-phenomenal activity and phenomenal content [21,22,23,44] (see below).

## 2. Neuronal Mechanisms—From Pre-Stimulus to Stimulus-Induced Activity

### 2.1. Pre-Stimulus Activity—‘Temporo-Spatial Context’ and ‘Temporo-Spatial Background’ of Stimulus-Induced Activity

One of the hallmark features of TTC is that it takes a more detailed view of brain neural activity. IIT and several other theories focus mainly on stimulus-induced activity and its distinct measures as neural correlates of consciousness [25]. In a nutshell: if we understand stimulus-induced activity by finding the ‘right’ measure, we will find a comprehensive neuroscientific explanation of consciousness. This assumption is not shared by TTC: Stimulus-induced activity is conceived in TTC as just the ‘tip of the iceberg’. This means that we need to search for what happens prior to stimulus-induced activity, its deeper layers, as those strongly shape whether the former can or cannot be associated with the phenomenal contents of consciousness.

What happens prior to stimulus-induced activity? This leads to the spontaneous or ongoing activity of the brain and, specifically, its pre-stimulus activity. Before tabulating existing assumptions and proposing new perspectives, we must first clarify the concept of ‘continuous brain activity’. Here we use the term ongoing activity to describe an amalgam that includes three distinct components, namely resting state, pre-stimulus, and stimulus- or task-related activity. The first component of ongoing activity concerns what is operationally described as the resting state, which is usually measured during the absence of specific stimuli or tasks, that is, closed or open eyes, for longer periods of time, that is, minutes [47]. Resting-state activity is often also described as intrinsic activity, which refers to the component of neural activity that is ongoing and not modulated by extrinsic tasks or stimuli and therefore present during both rest and task states [47,48,49,50].

Cognitively, resting-state activity has been associated with various forms of internally oriented cognition (see below for details). Given such an association of ongoing activity with cognition, one may consider the resting state or intrinsic activity as just another task state: it features covert internal stimuli or tasks not yet fully known rather than overt external tasks or stimuli as in what is typically described as task states [47,48,49,50,51].

The second component of ongoing activity can be found in pre-stimulus activity, that is, neural activity immediately preceding the onset of a specific stimulus or task, that is, 100–1000 ms before the onset of the stimulus [52,53,54,55,56,57]. This can be coined as the ‘temporo-spatial context’ as it precedes stimulus-induced activity but provides a temporo-spatial context for the latter. Finally, the third component of ongoing activity can be found during stimulus- or task-related activity, where it is present in what is also coined ‘temporo-spatial background activity’: it may represent the component of stimulus- or task-related activity that, operating in the background, reflects the temporo-spatial carry-over and continuation of the ongoing activity during task states or stimulation [48,49,50,58,59,60,61,62,63,64,65].

### 2.2. ‘Temporo-Spatial Context’ I—Additive Interaction of Pre-Stimulus and Stimulus-Induced Activity

Considering the previous section, we can distinguish wide and narrow views of stimulus-induced activity. A narrow view only focuses on those changes exclusively related to the stimulus itself. In contrast, a wider view conceives stimulus-induced activity as just one component of the ongoing activity of the brain. In that case, the pre-stimulus activity provides the temporo-spatial context and the background activity of stimulus-induced activity. TTC presupposes such a wider view, claiming that the background pre-stimulus activity that provides for stimulus-induced activity is key for yielding the phenomenal contents including their embedding or nesting within the ongoing stream of consciousness.

This raises two questions about purely neuronal issues. First, how does pre-stimulus activity as the temporo-spatial context interact with the actual input, stimulus, or task? Second, how can we measure the background activity (as carried over from pre-stimulus activity) during stimulus-induced activity? Let us address the first question.

Traditionally, neuronal activity related to external input/stimulation is supposed to be ‘added’ or superimposed on the ongoing level of pre-stimulus activity [66,67,68]. Importantly, the degree or magnitude to which the external input/stimulus elicits an amplitude in the post-stimulus interval is supposed to remain independent of the pre-stimulus activity level, that is, whether the latter is high or low (this is methodologically, for instance, reflected in the fact that the activity at stimulus onset is often set to zero, i.e., baseline correction, as in both EEG and fMRI). This amounts to a purely additive pre-stimulus-stimulus interaction [52,56,66,67]. The contents of consciousness are here conceived to remain independent of pre-stimulus activity levels. The TTC rejects such independence of the contents of consciousness as empirical evidence shows a strong dependence of post-stimulus activity on pre-stimulus activity; this shall be discussed in the next section.

### 2.3. ‘Temporo-Spatial Context’ II—Non-Additive Interaction of the Pre-Stimulus and Stimulus-Induced Activity

The assumption of additive pre-post-stimulus interaction stands counter to various fMRI studies [69,70,71] and EEG/MEG [55,72,73,74,75,76] that show the importance of pre-stimulus activity changes for conscious contents (see also [77]).

The impact of the pre-stimulus activity on stimulus-induced activity is mediated by specific neuronal features. For instance, high degrees of pre-stimulus variance [78,79,80], strong slow frequencies [81], or high synchronization [82] make it possible for the stimulus to induce larger changes, such as variance suppression with TTV reduction, shifts to faster frequencies, or desynchronization during the post-stimulus interval, which all make consciousness of the stimulus more likely. If, in contrast, pre-stimulus dynamics exhibits low variance, stronger faster frequencies, and low synchronization, the external stimulus can no longer induce much change, which decreases the chance of consciousness.

Given such a differential impact of low and high pre-stimulus activity levels (in distinct neurophysiological measures), the assumption of a purely additive pre-stimulus-stimulus interaction is put into doubt [52,56]. The impact of the stimulus is not simply added to the ongoing pre-stimulus activity, rather there must be a non-additive interaction—or better a modulation—of the input/stimulus with the level of pre-stimulus activity.

**Figure 2:**
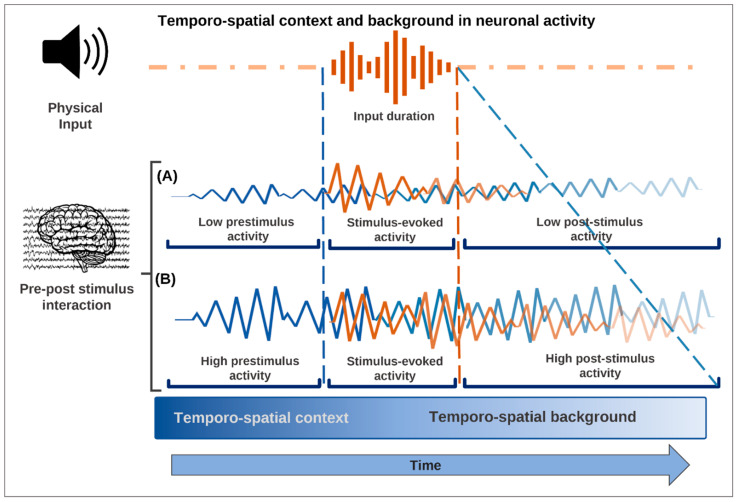
The temporo-spatial context precedes the stimulus-induced activity interacting with the input, while the temporo-spatial background represents that component of stimulus- or task-related activity that supports the persistence of the task or stimulus-related activity over time. (A) Low pre-stimulus activity is associated with little changes made by the stimulus in the neuronal activity and consequently with low post-stimulus activity making unlikely consciousness of the respective content. (B) Instead, high pre-stimulus activity makes it possible for the stimulus to induce larger changes in the neuronal activity, thus producing the temporo-spatial carry-over and continuation of the ongoing stimulus-related activity, which make it more likely that the stimulus will be perceived consciously.

Such a non-additive pre-stimulus-stimulus interaction is indeed confirmed by recent studies in fMRI [52], EEG/MEG [53,55,83,84], and cellular recordings [85]. The pre-stimulus activity provides a temporo-spatial context by means of which it can strongly shape the activity that the external input or stimulus can (or cannot) elicit, that is, stimulus-induced activity: the input effects are not simply added to the ongoing activity (Figure 2). The TTC assumes the non-additive component to be the key in associating consciousness to specific contents.

### 2.4. ‘Temporo-Spatial Background’—Trial-to-Trial Variability (TTV) of Stimulus-Induced Activity

How can we measure the impact of pre-stimulus activity during post-stimulus activity? One way is to measure trial-to-trial variability (TTV) that can be observed at both the levels of cellular and regional-systemic activity [78,85,86,87,88]. TTV measures the variation in the amplitude across different trials of one and the same stimulus. Given that the stimulus remains the same over all trials, the variation in the amplitude across trials can only be due to the carry-over of pre-stimulus activity levels to the post-stimulus interval. One can now measure the trial-to-trial variability during the post-stimulus interval relative to the pre-stimulus variability: one can typically observe a decrease in TTV around 100 to 500 ms during the post-stimulus period in EEG/MEG [52,53,55,78,84,88] and fMRI [52,56]. Hence, the ongoing variation in the amplitude of the pre-stimulus activity is carried over to the post-stimulus interval where it is transiently suppressed by the external input/stimulus.

Furthermore, recent studies in EEG/MEG [53,54,55,84] and on the cellular level [85] demonstrate that the level of post-stimulus TTV is strongly dependent on pre-stimulus activity. High levels of pre-stimulus activity lead to higher degrees of transient post-stimulus suppression than low levels of pre-stimulus activity. Such differential effects of low and high pre-stimulus activity on post-stimulus TTV again support the assumption of non-additive rather than additive pre-stimulus-stimulus interaction. Recent studies also show that the TTV exhibits a certain spatial and temporal pattern. Huang et al. [52] demonstrated that sensory and motor regions show lower degrees of TTV suppression than higher-order regions, such as default-mode network regions. Hence, there is a certain spatial topography of TTV changes that may depend on not yet fully clear intrinsic features of the regions themselves, such as their intrinsic neural timescales [36,37,38].

On the temporal-dynamic side, Wainio-Theberge et al. [53] demonstrate that TTV is related to low and high pre-stimulus activity levels in different ways in different frequency bands. A positive non-additive interaction with strong TTV reduction in especially lower pre-stimulus activity can be observed in the delta band (1–4 Hz), while a negative non-additive interaction with strong TTV reduction in higher pre-stimulus activity is observed in the alpha band (8–12 Hz).

Together, these findings suggest a distinct temporal dynamic of the way in which pre-stimulus activity is carried over to stimulus-induced activity when providing its temporo-spatial background (see also [38]). Pre-stimulus activity is thus carried over to and interacts with the post-stimulus interval in both spatial and temporal terms (Figure 2). We cannot consequently understand stimulus-induced activity without considering the carry-over effects from the pre-stimulus interval. This concerns their non-additive interaction as well as the carry-over of a distinct spatial topography and temporal dynamics from pre-stimulus to stimulus-induced activity. We will see now that these features are key to understanding how consciousness, including its specific phenomenal features, can be associated with contents.

## 3. From Pre-Stimulus to Stimulus-Induced Activity—Conscious vs. Unconscious Contents

### 3.1. Pre-Stimulus Activity—Prediction of Conscious vs. Unconscious Contents

We showed that, on purely neuronal grounds, pre-stimulus activity interacts with stimulus-induced activity in a non-additive way. Specifically, low and high pre-stimulus activity levels exert a differential impact on subsequent stimulus-induced activity. If such non-additive interaction is relevant for consciousness, one would expect that pre-stimulus activity levels should predict whether a subsequent stimulus or input is associated with consciousness. This is indeed supported by various studies that describe the impact of pre-stimulus activity levels on the contents and level or state, i.e., arousal of consciousness in fMRI, EEG/MEG, or single-unit activity. Regarding the content, fMRI studies showed that if the level of pre-stimulus activity in the fusiform face area (FFA) is high, subjects will perceive the stimulus as a face, while low pre-stimulus activity in FFA will bias subjects towards perceiving the same stimulus as a vase [69,70,71,89]. Hence, it is the spatial (FFA) and temporal (increase in FFA prior to stimulus arrival) context provided by the pre-stimulus activity that shapes your post-stimulus activity, including its phenomenal contents.

The pre-stimulus power and phase synchrony of alpha oscillations (8–13 Hz) in the EEG strongly impacts whether one perceives a visual stimulus or not [55,72,74,76]. Other frequencies in the pre-stimulus period, such as slow cortical potentials (0.1 to 1 Hz) and delta, theta, and beta power, but also pre-stimulus functional connectivity (e.g., the correlation between different regions’ time-series), impact subsequent stimulus-related activity and its association with consciousness [69,70,72,75,81]. Single-cell recordings in monkeys showed that the level and amount of synchrony of spontaneous V1 activity determined whether subsequent stimulus processing would engage recurrent processing and hence conscious perception and report of the stimulus [82]. Furthermore, a high degree of synchronization in the pre-stimulus state is associated with higher degrees of post-stimulus desynchronization, which enhances awareness of the respective stimulus or content [90]. Together, these data suggest that consciousness is dependent not only on the stimulus-related activity itself but also on the pre-stimulus level of activity. This effect seems to arise primarily by how the latter (pre-stimulus activity) shapes the former (stimulus-induced activity).

In addition to predicting specific contents of consciousness, another role for pre-stimulus activity may be that it reflects the level of arousal, i.e., the conscious state [91,92]. It has been argued that pure processing of content is itself not sufficient for consciousness and that a certain level or arousal must also be associated with that content [21,93,94,95]. In that case, one would expect that pre-stimulus activity serves a dual role, that is, mediating both content and its associated level or arousal.

A recent MEG study tested such a dual role, indeed demonstrating that pre-stimulus activity reflected both category-specific content of consciousness (as pre-stimulus activity levels predicted subsequent contents) and a more general process related to arousal (as indexed by pupil size) [75]. Specific temporal dynamic features like alpha power or others, as well as spatial topography like Fusiform Face Area (FFA) activity during the pre-stimulus activity period, can predict whether a subsequent input or stimulus can (or cannot) become conscious. This suggests a strong impact of the pre-stimulus activity’s temporo-spatial context on conscious contents as related to the stimulus-induced activity.

Two features remain open, though. The first are the mechanisms by means of which pre-stimulus activity impacts conscious contents as measured during stimulus-induced activity, since studies probing for non-additivity in a direct way during conscious content remain yet to be reported. Second, the key relevance of pre-stimulus activity for the phenomenal features of consciousness remains yet unclear. We will discuss in the third part how the role of pre-stimulus activity as a temporo-spatial background may be key in constituting the phenomenal features of consciousness.

### 3.2. Post-Stimulus Activity—TTV Predicts Conscious vs. Unconscious Contents

We identified post-stimulus TTV as a marker of the degree to which pre-stimulus activity is carried over to the post-stimulus interval providing the temporo-spatial background (see above 2.3). This has indeed been shown especially for the case of visual perception: the more strongly the post-stimulus TTV is reduced (for instance, in DLPFC and/or the alpha band), the more likely it is that the respective contents will become conscious [72,73,74,78,80,88,96,97,98,99]. These findings strongly suggest that post-stimulus TTV plays a key role in mediating conscious contents, which, albeit indirectly, implies the presence of non-additive rather than additive pre-post-stimulus interactions. Sergent et al. [100] demonstrated that the level of post-stimulus TTV around 250–300 ms provides a critical threshold: after that, stimulus-related activity may persist and thus be prolonged, showing low TTV during conscious access. If, in contrast, the TTV returns to its high pre-stimulus level during the post-stimulus period, conscious access is no longer present [100]. The critical role of 250–300 ms post-stimulus is very compatible with a recent intracranial stereo-electroencephalography (sEEG) (with a no-report paradigm) that showed the differential impact of the pre-stimulus activity on early and late post-stimulus activity: pre-stimulus variance more strongly shaped the early (0–250/300 ms) than the late (300–600 ms) post-stimulus interval where the impact of the external stimulus was stronger [84], which may provide conscious access [100].

Accordingly, it is the degree of change during post-stimulus activity relative to the intrinsic ongoing dynamics of the pre-stimulus interval, as measured by the TTV, which seems to be central for associating consciousness to external stimuli. Taken in such a sense, consciousness can neither be associated exclusively with either post-stimulus activity nor pre-stimulus activity. Instead, the pre- and post-stimulus activity must interact in a certain way, i.e., non-additively, with supposedly strong TTV quenching, i.e., stimulus-related suppression of the ongoing pre-stimulus variability, to associate consciousness to contents. Thus, as TTC argues that the pre-stimulus activity is of fundamental importance for the formation of the content of consciousness (not a mere influence element), at the same time, the post-stimulus phase is also relevant although more difficult to define.

Recent studies provide a possible answer to the role of intrinsic neural timescales in inferring future inputs; with an empirical prior and prediction error, based on the temporo-spatial carry-over and continuation of ongoing activity within the post-stimulus interval, the task states during which the respective content is consciously experienced [39,40]. In this regard, the prediction error is the difference between the ongoing (from pre-stimulus to stimulus period) relevant timescale in the primary sensory area (e.g., a short timescale) to the related timescale of the input at a discrete point in time (e.g., a musical note). The timescales of the input at its discrete point in time are integrated within the ongoing timescales of the respective sensory region, such as the auditory cortex in this case. This is additionally modulated by the longer timescales of higher-order associative regions that provide top-down predictions to lower-order areas, while receiving prediction error signals from lower-order areas (i.e., the difference in timescales between the input and sensory area) [40]. Thus, post-stimulus activity is not only relevant to the temporal modeling of the input but also provides by itself a temporal extension of the input by the longer timescales of the higher-order regions through their top-down modulation and prediction. Therefore, pre-stimulus and post-stimulus activity are functionally separate only in relation to the single stimulus, while on a larger timescale, such as that of the stream of consciousness, the two neural mechanisms are just distinct temporal scales that are integrated and connected within the ongoing neuronal activity stream, which, as we assume, relates to the ongoing stream of consciousness, e.g., especially its transitive parts. Finally, it shall be mentioned that we recently provided a first attempt to describe the pre-post-stimulus relationship in mathematical terms and that the use of category theory allowed us to formalize the pre-post-stimulus relationship explicitly in relational terms [43].

## 4. From Pre-Stimulus to Stimulus-Induced Activity—Neuro-Phenomenal Mechanisms

It has been shown that to describe and predict the presence of the phenomenal contents of consciousness, the stimulus-induced activity is not sufficient and the pre-stimulus activity and its relationship with stimuli are also necessary. Specifically, the temporo-spatial context refers to the impact of the pre-stimulus activity on the post-stimulus activity, thus increasing or decreasing the probability that the stimulus will be consciously perceived. By contrast, the temporo-spatial background refers to how pre-stimulus activity is carried over to stimulus-induced activity, shaping the phenomenal content of the received stimulus. How do these two neuronal features relate to phenomenal features? Let us start with the temporo-spatial context.

### 4.1. Temporo-Spatial Context and Pre-Phenomenal Experience

The temporo-spatial context on the neuronal level implies that the phenomenal content of each stimulus is not independent of what preceded it; indeed, how these contents emerge at the level of consciousness strongly depends on the context in which we are immersed. In other words, we can conceptualize the temporo-spatial context as a neural analogue of what has been described phenomenologically as ‘horizon-intentionality’ [18,94,101,102], that is, the pervading activity that temporally constitutes and frames the possible next experiences based on the previous ones (as distinguished from the actual experience itself). Since we are usually not fully and directly conscious of such a horizon intentionality, one may want to characterize it as a ‘pre-phenomenal’ component of the ongoing stream of consciousness (which is similar to what James called the ‘transitive part’ of the stream [33]): it shapes what kind of possible phenomenal contents can occur in consciousness but is not experienced as such in phenomenal terms; see [44] for the notion of ‘pre-phenomenal’). The pre-phenomenal factor is strictly related to the two proposed additional timescales, which provide the transitive part of consciousness that temporally links the different contents together. It is an empirical specification of the temporo-spatial context that influences the way the stimulus is perceived (consciously or unconsciously), without, however, being a direct object of consciousness, which is instead associated with shorter timescales, as proposed by IIT. Hence, it is a pre-phenomenal factor instead of a phenomenal one but still different from a purely non-phenomenal factor (i.e., something completely extrinsic and independent from experience) [44].

**Figure 3:**
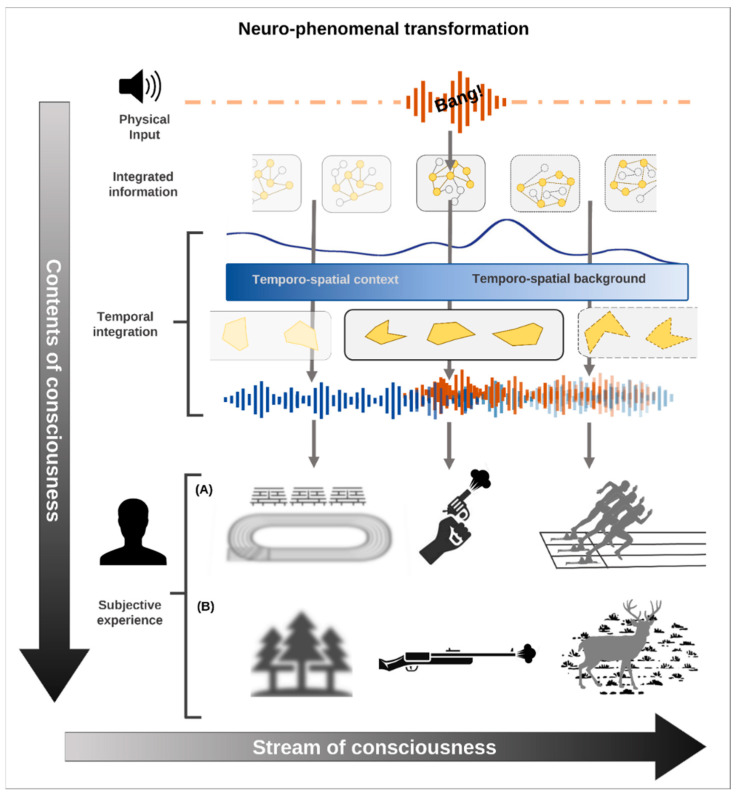
The vertical axis represents the discrete contents of consciousness, while the horizontal axis represents the ongoing stream of consciousness that embeds single phenomenal contents within a sequence or series of phenomenal contents. On a neuro-phenomenal level, with the same sound inputs (the ‘bang’ at the top) and the same kind of post-stimulus information integration, two different contents of consciousness can be experienced depending on the type of temporal integration between pre- and post-stimulus activity. Scenario (A): The ‘bang’ will be perceived as a starting gun connecting the temporo-spatial context associated with the stadium and the temporo-spatial background associated with the runners. Scenario (B): the ‘bang’ will be perceived as a starting gun that will link the temporo-spatial context associated with the forest and the temporo-spatial background associated with the deer.

For example, the sound produced by a firearm produces different phenomenal contents depending on the temporo-spatial context. Imagine that you heard a gun bang that constitutes your stimulus onset. Prior to that—the pre-stimulus activity—you were seeing or thinking about a stadium: in that case, you experience the ‘bang’ as the start of a 100-m race in the stadium. If, in contrast, you were in the middle of a forest or you were thinking about your last walk through the forest during the hunting season, you may experience the ‘bang’ in relation to a hunter shooting down a deer. Consequently, the actual phenomenal content of your consciousness during the post-stimulus period is biased by and thus depends on your pre-stimulus period and its range of possible biasing phenomenal contents (Figure 3). The TTC thus speaks of the temporo-spatial expansion: pre-stimulus activity and its associated pre-phenomenal contents are carried over into the post-stimulus consciousness featured by its phenomenal contents. Let us detail that carry-over.

The carry-over allows expansion of the pre-stimulus activity with its range of possible pre-phenomenal contents (as defined by the pre-stimulus activity’s spatial and temporal features) into the post-stimulus period. Here they interact in a non-additive way with the actual input and thereby shape the actual phenomenal content of consciousness. The TTC therefore speaks of temporo-spatial expansion on both neuronal and phenomenal levels: the pre-stimulus activity is expanded into the stimulus-induced activity, which makes possible the expansion of the possible pre-phenomenal contents into the actual phenomenal content itself.

### 4.2. Temporo-Spatial Background and Phenomenal Experience

This leads us to the second feature, the temporo-spatial background against which or within which the actual input is processed. If the actual input interacts with the temporo-spatial background in a non-additive way, it will be assigned consciousness—the content is processed and therefore perceived or experienced. Otherwise, the content will be processed but not perceived/experienced.

Why is the non-additive interaction so important for consciousness? We do not simply experience consecutive or juxtaposed contents at discrete points in time. Instead, every single phenomenal content of consciousness is embedded and nested within the ongoing subjective experience, i.e., the stream of consciousness including its various phenomenal contents. This embedding of a single phenomenal content within the ongoing stream of conscious contents is, as we assume, neuronally related to the temporo-spatial background during the post-stimulus period: the latter allows integration of the inputs into the ongoing activity’s temporal stream and spatial topography.

Taken together, the IIT is right in highlighting the relevance of integration. Converging IIT with the TTC, we postulate that what IIT so well describes as information integration can be specified as temporo-spatial integration. The actual input, including its actual spatiotemporal features, such as duration and extension, is integrated and embedded within the ongoing temporo-spatial background of the brain’s stimulus-related activity. One can thus speak of temporo-spatial integration, which, in turn, provides the ground for integrating different contents including their information. This suggests that information integration, as per IIT, can be well converged with the temporo-spatial framework as assumed by the TTC.

Taken in a more phenomenal sense, the temporo-spatial background allows one to put the input/content within the context of the temporo-spatial features of the ongoing activity. The duration of the temporo-spatial background expands beyond that of the actual input/components, and the same holds for the spatial extension. If now the actual input/content is integrated and thus interacts non-additively with the temporo-spatial background, the duration and extension of the former are expanded both in time and space. That temporo-spatial expansion, in turn, is key for the content to become conscious: the longer the expanded duration and the more expanded the spatial extension, the more likely consciousness will be assigned to the input/content. This makes possible what is phenomenologically described as object-intentionality [101].

## 5. Conclusions

We started with the axioms of IIT that describe the constitution of the phenomenal content of consciousness. This was followed by pointing to three types of temporal integration: integration between inputs into a content, integration of content with other contents, and integration of phenomenal contents within the ongoing stream of consciousness. Following its predictions, IIT focuses on the short-term timescales of the first type of integration, while more or less neglecting the longer-term timescales of the second and third forms of temporal integration. To close this temporal gap, we propose converging the IIT with the TTC, the Temporo-spatial Theory of Consciousness, as that takes into view multiple timescales including both short- and long-term. These long-term timescales not only correspond to experiences of longer duration but also provide continuity and unity in the background of different experiences.

The TTC assumes temporo-spatial expansion as a key mechanism for the interaction of pre-stimulus and post-stimulus activity, which it deems key for constituting phenomenal content. Specifically, the pre-stimulus activity provides the temporo-spatial context, which allows for the third type of temporal integration, the integration of phenomenal content within the ongoing stream of consciousness. At the same time, the pre-stimulus activity overlaps into a stimulus-induced activity where it provides the temporo-spatial background—this is central for the second kind of temporal integration, the integration between different phenomenal contents.

In conclusion, temporo-spatial expansion (as one of the four mechanisms postulated in TTC) provides the neuronal mechanism for the second and third types of temporal integration operating on longer timescales (300 ms–3 s, >3 s). This would complement IIT with its focus on the first type of temporal integration featured by short timescales (100–300 ms). More generally, TTC provides a larger and temporally more comprehensive framework for IIT (and other neuroscientific theories; [25,103])—the latter concerns short-term temporal aspects of consciousness that can be complemented by the longer-term temporal features and mechanisms of the temporo-spatial framework provided by TTC. This makes it possible to also consider yet another key feature of TTC, namely, the assumption that temporo-spatial features are shared by neuronal and phenomenal states as their ‘common currency’ [23,104,105], which, in part, converges with the assumption of their identity in IIT.

Essentially, the concept of ‘common currency’ refers to features shared by both neural and mental features; the TTC considers these shared features to consist in spatial topography and temporal dynamic. Consciousness itself and especially its phenomenal features are thereby considered in primarily temporal and spatial terms, hence the concepts of mental topography and mental dynamic.

We must be careful on conceptual grounds: shared features in terms of ‘common currency’ do not necessarily imply that neuronal and mental (or phenomenal) features are identical. This is an additional and more far-reaching claim that extends beyond the assumption of ‘common currency’, which, therefore, does not entail identity. That distinguishes TTC from IIT in that it assumes structural identity between the phenomenal properties of a particular experience and the information/causal properties of a conceptual structure specified by a PSC [1,2,3,106,107]. Conversely, identity is only possible if there are shared features such that identity (as claimed by IIT) does indeed entail common currency (as postulated in TTC) but not vice versa.

## Data Availability

Not applicable.
